# Achieving a healthy indoor environment by using an emissions barrier to stop the spread of chemicals from a building into the indoor air

**DOI:** 10.14324/111.444/ucloe.000033

**Published:** 2022-03-17

**Authors:** Lennart Larsson, Johan Mattsson

**Affiliations:** 1 Lund University, Lund, Sweden; 2 cTrap AB, Råbylunds Gård, Prästavägen 12, 224 80 Lund, Sweden

**Keywords:** emissions barrier, adsorbent, healthy buildings, restoration

## Abstract

An emissions barrier was used in a premises due to complaints about the indoor air quality (IAQ) as a result of emissions from the building in question. The emissions comprised chlorophenols/chloroanisoles and polycyclic aromatic hydrocarbons (PAH) from treated wood and volatile organic compounds (VOCs), mainly 2-ethylhexanol, from polyvinyl chloride (PVC) flooring and the glue used to paste the flooring onto a concrete slab. Attaching the barrier at the surfaces from where the emissions were spread (floor, walls, ceilings) resulted in a fresh and odour-free indoor air. We conclude that using an emissions barrier in buildings made unhealthy by moisture is an efficient way of restoring pleasant and healthy indoor air.

## Introduction

Building moisture typically results in the spread of chemical and biological emissions into the indoor air leading to illnesses and symptoms such as asthma, skin and eye irritation, fatigue, etc. [1,2]. Drying is a necessary first step in remediation of a building as it will stop further moisture-driven reactions with the building materials as well as (continued) mould growth. However, drying is not enough to secure clean indoor air, as the numerous chemicals that have been formed from water – or moisture – acting on the materials will still remain in the building, and over time will inevitably be emitted into the indoor air. The emissions may be, for example, volatile organic compounds (VOCs) from paints, glue, insulation materials, chipboard, microorganisms, impregnation and plasticiser chemicals, or toxins from microorganisms such as mould (see, e.g., [3]).

Airborne particles released during building construction may be removed by using portable air cleaners with mechanical air filtration, for example, with high efficiency particulate arresting (HEPA) filters or by electronic cleaning whereby the particles are charged and thereafter accumulated on a collector or precipitated following a reaction with ions generated with an ion generator [4]. VOCs (including odours) may be removed by pumping the air through a filter containing an adsorbent. Some air cleaners are designed to destroy the contaminants; for example, microbes may be killed by ultraviolet (UV) light. Photocatalytic oxidation (PCO) cleaners and ozone generators use UV together with a catalyst to convert harmful pollutants to less harmful products. Such measures, as well as increasing the ventilation, may decrease the concentrations of the airborne contaminants, but will not prevent them from being spread into the indoor air. Furthermore, PCO cleaners [5] as well as ozone generators [6,7] may in fact increase the concentrations of some other VOCs including potential lung irritants such as formaldehyde. Replacing damaged materials with new ones may, in some instances, be useful but also very time-consuming, costly and – when the damaged materials are vital for the stability and function of the building – often impossible to do.

Attaching a sealant on indoor surfaces (floors, ceilings or walls) from where the emissions are spread constitutes an alternative approach. Examples of sealants are various polymers, aluminium/plastic laminates, etc. Such sealants can be extremely efficient in stopping the emissions and thus improving the indoor air quality (IAQ); however, it is necessary to first know the source of the emissions. In the present study we applied a new type of emissions barrier [8,9] developed at Lund University, Sweden to stop emissions in some buildings with different complaints regarding the IAQ.

## Methods

Three buildings with IAQ complaints due to emissions resulting from the construction of the building were studied. In short, the surfaces from where the emissions were spread (floors, ceilings, walls) were covered with an emissions barrier to prevent them from reaching the indoor air. In the specific barrier used, the cTrap, an adsorption layer functions together with a hydrophilic polymer sheet making the adsorption virtually irreversible [8,9]. The flexible cTrap cloth was attached to the surfaces using an adhesive tape and/or a staple gun. The indoor air concentrations of the emissions were measured both before and after the cTrap installations by pumping air through an adsorbent material followed by thermal or chemical desorption and analysis using gas chromatography-mass spectrometry by Eurofins Pegasuslab Ltd, Uppsala, Sweden.

## Results

1. We studied the living room and a bedroom of a wooden summer house built in 1964 with a disturbing ‘summer cottage smell’ indoors which was attributed to chloroanisoles. The building had previously been treated with chlorophenol-containing preservatives which were widely used in the 1960s and 1970s; in moist conditions chlorophenols may be biomethylated to form chloroanisoles having an intense, characteristic mould-like odour. The ceiling, walls and floor in the bedroom (as well as the doorway between the bedroom and the living room), but not in the living room, were covered with the cTrap cloth. Subsequently, air sampling for chlorophenols/chloroanisoles was carried out simultaneously in both rooms. Tetrachlorophenol, trichloroanisole and pentachloroanisole were detected in the air of the living room, but only tetrachlorophenol was found in the bedroom, and in an air concentration 93% lower than in the living room (Table 1). In addition, the cTrap installation team reported the disappearance of the mouldy odour in the bedroom.

**Table 1.** Results of cTrap installations (μg/m^3^ air)

Emissions	Without cTrap	With cTrap
Tetrachlorophenol	0.14	0.01
Chloroanisoles	0.013	n.d.
PAH	1.726	0.139
2-ethylhexanol	63	1.5

n.d., not detected (<0.0018 μg/m^3^ 2,4,6-trichloroanisole; <0.00091 μg/m^3^ pentachloroanisole).

2. A building where a creosote-based tar layer had been attached to the concrete slab as a moisture barrier was studied. The air concentrations of polycyclic aromatic hydrocarbons (PAH) were 1726 ng/m^3^ air. There was a disturbing smell similar to that of car tires or asphalt inside the building which persisted even after the tar had been removed. The cTrap cloth was installed on about 75% of the wall surface. The smell disappeared and the PAH air concentrations decreased to 139 ng/m^3^, thus corresponding to a reduction of 92% (Table 1).

3. A townhouse was studied where the tenants suffered from itching all over their bodies while at home, symptoms which disappeared when they were outside the building. A polyvinyl chloride (PVC) flooring had been glued onto a concrete slab which had become moist through the diffusion of water from the ground. The air concentration of 2-ethylhexanol, a compound which is ubiquitous in small concentrations in indoor air but which was found in increased concentrations, for example, following hydrolysis of the glue and/or phthalates of PVC floorings, was found to be 63 μg/m^3^ (directional measurement). The cTrap was attached to the existing flooring, and the itchiness disappeared. Three months after the cTrap was installed the air concentration was 1.5 μg/m^3^ (Table 1), a value which persisted in a follow-up study six years after its installation; the residents have reported no further symptoms.

Results are summarised in Table 1.

## Discussion

Staying in a moist building can cause health problems [1,2], for example, skin and mucous irritation and respiratory disorders. Such conditions are frequently referred to as building-related illnesses (BRI) and are caused by the spread of chemical emissions from the building itself into the indoor air. Research has shown that mixtures of VOCs emitted from building materials may act in synergy in worsening the perceived air quality [10,11] even when present in concentrations below the odour thresholds. The studies presented here demonstrate that such emissions can be effectively stopped by using an emissions barrier. Through scientifically validated questionnaires it has also been found that health symptoms and/or unpleasant odours can be decreased or totally eliminated (data not shown). Still, the awareness that use of emissions barriers is an efficient weapon against BRI is not widespread – not even among indoor air professionals.

The specific device used in the studies presented here, the cTrap (surface emissions trap) cloth, contains two active layers; one adsorption layer and one hydrophilic polymer layer. The device is airtight while at the same time allowing moisture to pass through with almost no resistance at all, and will thus not affect the moisture balance of the building. It has been shown to be efficient against a wide range of chemicals including, for example, alcohols, aldehydes, sulfur compounds, PAH, chloroanisoles, chlorophenols, mould products and odours [8,9], and the effect has been found to be long term (several years). After the barrier has been applied to a floor, a surface layer, for example, a laminate, parquet or plastic flooring, etc., is installed on top of the cTrap cloth. When attached on walls or to a ceiling the cloth is usually covered with a gypsum board which is then painted or decorated with a wallpaper. When the building is eventually demolished the cTrap cloth, with the adsorbent layer containing the emitted chemicals, will be disposed of by combustion thus avoiding leakage of the chemicals into the environment from deposited building materials.

In summary, we conclude that use of an emissions barrier represents an effective, economic, and eco-friendly way of restoring healthy indoor air in buildings affected by moisture.

## Conclusions

An emissions barrier can be used to restore fresh and healthy indoor air in buildings made unhealthy due to moisture damage.

## Data Availability

No further data was used in addition to referenced works.
